# Fibroblast Growth Factor Receptor 1-4 Genetic Aberrations as Clinically Relevant Biomarkers in Squamous Cell Lung Cancer

**DOI:** 10.3389/fonc.2022.780650

**Published:** 2022-03-25

**Authors:** Joanna Moes-Sosnowska, Joanna Chorostowska-Wynimko

**Affiliations:** Department of Genetics and Clinical Immunology, National Institute of Tuberculosis and Lung Diseases, Warsaw, Poland

**Keywords:** fibroblast growth factor receptor, FGFR inhibitors, squamous non-small cell lung cancer (Sq-NSCLC), molecular biomarkers, targeted therapy, FGFR1, FGFR2, FGFR3

## Abstract

Fibroblast growth factor receptor (FGFR) inhibitors (FGFRis) are a potential therapeutic option for squamous non-small cell lung cancer (Sq-NSCLC). Because appropriate patient selection is needed for targeted therapy, molecular profiling is key to discovering candidate biomarker(s). Multiple FGFR aberrations are present in Sq-NSCLC tumors—alterations (mutations and fusions), amplification and mRNA/protein overexpression—but their predictive potential is unclear. Although FGFR1 amplification reliability was unsatisfactory, *FGFR* mRNA overexpression, mutations, and fusions are promising. However, currently their discriminatory power is insufficient, and the available clinical data are from small groups of Sq-NSCLC patients. Here, we focus on FGFR aberrations as predictive biomarkers for FGFR-targeting agents in Sq-NSCLC. Known and suggested molecular determinants of FGFRi resistance are also discussed.

## Introduction

Lung cancer is the most common cause of cancer-related death worldwide (1). Non-small cell lung cancer (NSCLC) accounts for over 75% of all lung cancer cases, and 20–30% are squamous non-small cell lung cancers (Sq-NSCLC). The prognosis of patients with advanced Sq-NSCLC is poor and immune checkpoint inhibitors (pembrolizumab, atezolizumab, or cemiplimab in tumors with PD-L1 expression≥50%; pembrolizumab plus carboplatin/nab-paclitaxel chemotherapy or nivolumab and ipilimumab plus 2 cycles of platinum-based chemotherapy regardless of PD-L1 expression) are the only first line systemic therapies approved by both the FDA (US Food and Drug Administration) and the EMA (European Medicines Agency) (2). However, in a randomized trials, approximately 30-40% of patients responded to first-line checkpoint inhibitors (2, 3). It is important to identify new targeted therapies and reliable predictive molecular biomarkers for Sq-NSCLC. Since, FGFR aberrations have been found in targetable oncogenic pathways (4), the FGFR inhibitors emerged as potential targeted therapy agents with promising therapeutic effects assessed in distinct clinical trials [reviewed in (5–7)]. Accordingly, in April 2019 the first FGFR inhibitor (FGFRi), erdafitinib, was approved by the FDA for patients with locally advanced or metastatic urothelial carcinoma with *FGFR*2 and *FGFR3* genetic alterations. In April 2020, the *FGFR1/2/3* inhibitor pemigatinib was granted approval and recently, in May 2021, the pan-FGFR1–4 inhibitor BGJ398 (infigratinib) was approved for the treatment of unresectable locally advanced or metastatic cholangiocarcinoma with *FGFR2* fusion or other rearrangements.

Sq-NSCLC is a target for FGFRi therapy because of the high rate of amplifications in fibroblast growth factor receptor 1 (*FGFR1*). Accordingly, FGFR1 amplification and overexpression are promising predictive markers for FGFRi therapy in Sq-NSCLC. However, most early phase clinical trials with new FGFRis showed only a partial response (8–11).

Appropriate patient selection is needed for targeted therapy. This review focuses on *FGFR* aberrations as reliable predictive biomarkers for response to FGFRis in Sq-NSCLC.

## FGFR Signaling Pathway—Structure and Normal Function of Fibroblast Growth Factor Receptors

**Figure 1:**
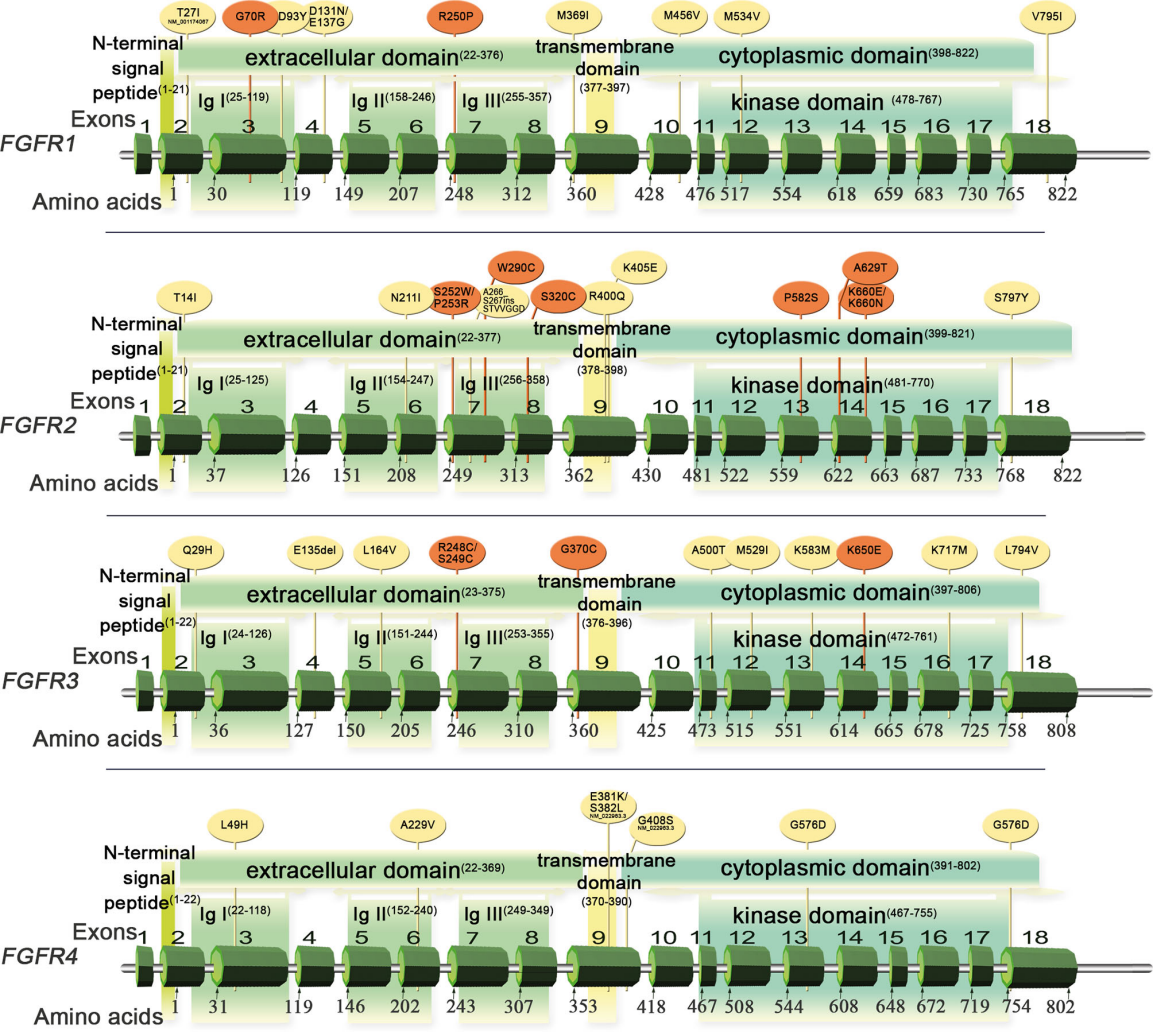
Schematic of FGFR1–4 genes and protein structures with variants detected in Sq-NSCLC tissue (**Table 1**). Red, pathogenic or likely pathogenic clinical significance; yellow, variants not in the ClinVar database.

**Figure 2:**
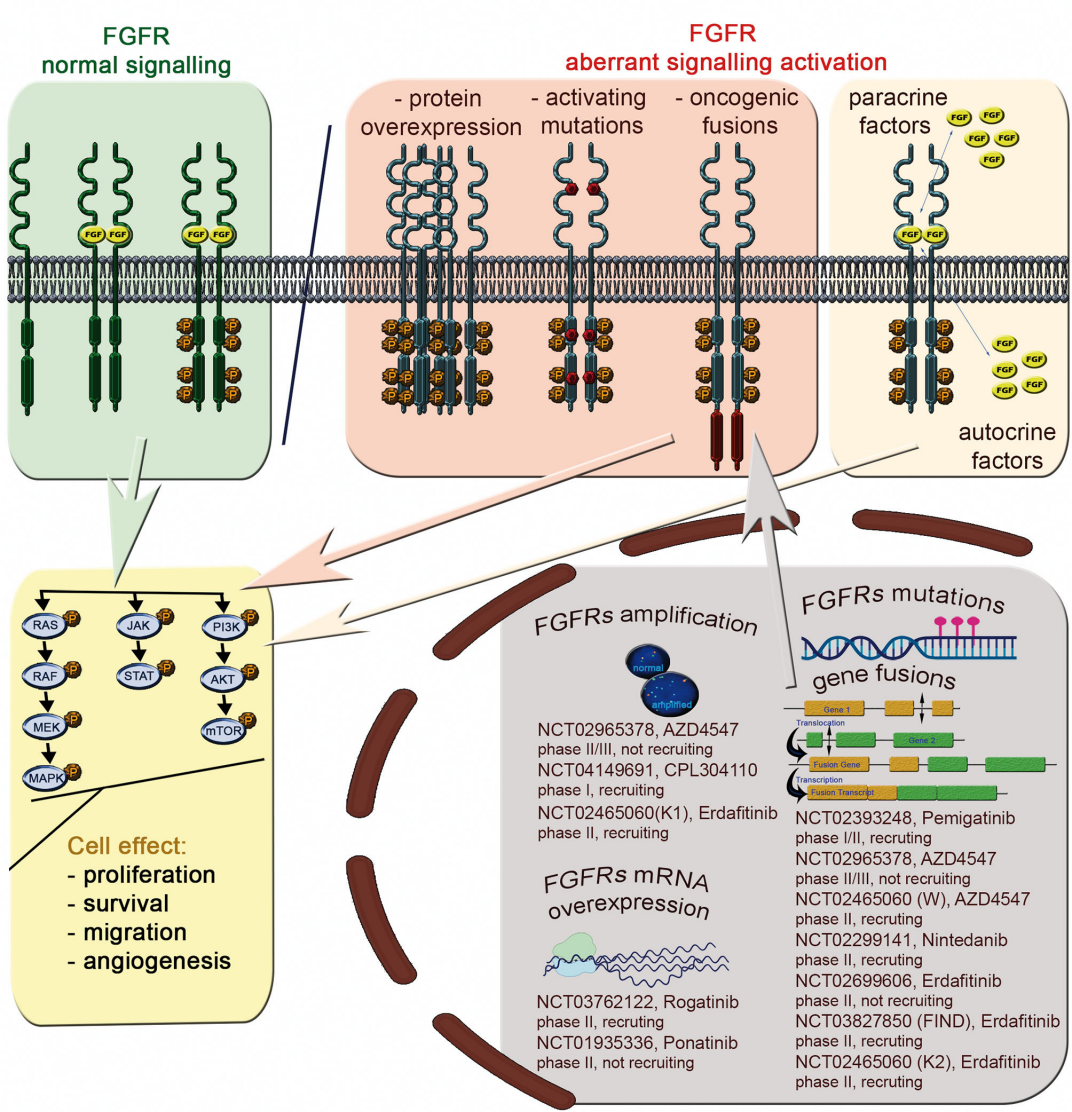
The FGFR signaling pathway and aberrations with potential as predictive biomarkers for FGFRi treatment of Sq-NSCLC.

*FGFR1-4* are located on four chromosomes and consist of 18 exons (start codon in exon 2). The proteins encoded by *FGFR* genes are members of the fibroblast growth factor family, consisting of 18 ligands acting *via* four highly conserved tyrosine kinase receptors (FGFR 1–4). Each FGFR consists of an extracellular region, composed of three immunoglobulin-like domains (IgI–IgIII), a single hydrophobic transmembrane domain, and a cytoplasmic tyrosine kinase domain (**Figure 1**, **Supplemental Figure S1** and **Table S1**). *FGFR*s have multiple alternative splice isoforms with tissue-specific expression (**Figure S1**). These are generated by deletion of the IgI domain and/or acid box or by a sequence change in the carboxy-terminal half of the IgIII domain, transforming isoform IIIb into IIIc (FGFR1–3) (12, 13). The extracellular domains interact with fibroblast growth factors (FGFs): FGFR1 binds both acidic and basic FGF; FGFR2 binds acidic, basic, and/or keratinocyte FGFs, depending on the isoform; FGFR3 binds acidic and basic FGFs; and FGFR4 binds acidic FGFs (**Supplemental Table S1**) (14). FGF ligand binding leads to dimerization of FGFR followed by its activation by sequential autophosphorylation of tyrosine residues (**Figure 2**). FGFR signaling activates the phospho-inositide-3-kinase (PI3K)/AKT, signal transducer and activator of transcription (STAT), and mitogen activated protein kinase (MAPK) pathways (15). FGFRs participate in the regulation of multiple biological activities, including tissue repair; angiogenesis; and cell proliferation, differentiation, migration, and survival (14–16). The four FGFR proteins are expressed in a number of tissues under normal conditions (**Table S1**). Aberrations of *FGFR1–4* genes are associated with a broad range of developmental disorders, such as craniosynostosis and dwarfing syndromes, and with cancers (15).

## FGFR1-4 Genetic Aberrations in Cancers

Deregulated FGF/FGFR signaling associated with FGFR aberrations is observed in various cancers (**Supplemental Table S2**), including lung cancer (**Table 1**). Activation of effectors of FGFR cancer-related signaling pathways (PI3K/AKT, STAT and MAPK) affects cell proliferation, survival, metabolism, migration, and cell cycle. Furthermore, FGFs released from tumor and stroma may increase autocrine and paracrine signaling and initiate angiogenesis (13) (**Figure 2**).

**Table 1:** Frequencies of *FGFR1–4* amplifications, mutations, and fusions in lung cancer.

Gene name	Type of Cancer	Amplificationfrequency (%)	Frequency of mutations (%)	Frequency of fusions (%)	Source
**FGFR1**	NSCLC	7.4% (2/27)	na	na	(8)
	Sq-NSCLC	22% (34/153)	0% (0/94)	na	(17)
		22% (22/101)	na	na	(18)
		14.4% (13/90)	na	na	(19)
		10.7% (8/75) 13.3% (10/75)	2.7% (2/75)	0% (0/75)	(20)
		11% (11/100)	na	na	(21)
		9% (ns/93)	0% (0/93)	0% (0/93)	(22)
		90% (9/10)	18% (2/11)	na	(23)
		25.2% (49/194)	na	na	(9)
		23% (37/156)	na	ns	(24)
		4.6% (6/130)	0% (0/130)	ns	(25)
		19.1% (30/157)	na	na	(26)
		19% (14/73)	na	na	(27)
		na	na	0.2% (1/492) BAG4-FGFR1	(28, 29)
		na	na	0.64% (2/312) BAG4-FGFR1	(30)
	Lung adenocarcinoma	5,7% (17/298)	na	na	(26)
		0% (0/77)	1% (1/94)	na	(17)
		na	na	0% (0/299)	(28)
		na	na	0% (0/492)	(29)
		na	na	0% (0/1016)	(30)
		ns	ns	0.005% (1/17827) BAG4-FGFR1	(31)
	Small-cell lung carcinoma	7.8% (6/77)	na	na	(32)
		5.6% (14/251)	na	na	(33)
		8% (3/37)	na	na	(24)
**FGFR2**	Sq-NSCLC	0% (0/75)	2.7% (2/75)	0% (0/75)	(20)
		0% (0/93)	3% (ns/93)	0% (0/93)	(22)
		na	0% (0/101)	na	(18)
		na	2.8% (5/179)	na	(34)
		na	4.7% (2/42)	na	(35)
		ns	ns	0.45% (1/222) FGFR2-KIAA1967	(28)
		na	na	0.2% (1/492) FGFR2-CCAR2	(29)
	Lung adenocarcinoma	na	na	0% (0/492)	(29)
		ns	ns	0.04% (8/17827)	(31)
		na	na	0% (0/299)	(28)
**FGFR3**	Sq-NSCLC	0% (0/75)	0% (0/75)	1.33% (1/75) FGFR3-TACC3	(20)
		5.5% (2/36)	0% (0/76)	5.3% (4/76) FGFR3-TACC3	(36)
		na	3.3% (6/179)	na	(34)
		na	9% (1/11)	na	(23)
		0% (0/93)	3% (ns/93)	0% (0/93)	(22)
		na	1% (1/101)	na	(18)
		ns	ns	1.92% (2/104) FGFR3-TACC3	(37)
		na	ns	4,16% (2/48) FGFR3-TACC3	(38)
		na	na	1.8% (4/222) FGFR3-TACC3	(28)
		na	na	0.6% (3/492) FGFR3-TACC3	(29)
		na	na	2.88% (9/312) FGFR3-TACC3	(30)
		ns	ns	0.58% (21/3582) FGFR3-TACC3	(31)
	Lung adenocarcinoma	ns	ns	0.06% (11/17827) FGFR3-TACC3	(31)
		na	na	0% (0/299)	(28)
		na	na	0% (0/492)	(29)
		na	na	0.59% (6/1016) FGFR3-TACC3	(30)
		0% (0/6)	0% (0/111)	1.8% (2/111) FGFR3-TACC3	(36)
		na	5.5% (20/363)	na	(39)
**FGFR4**	Sq-NSCLC	0% (0/75)	5.3% (4/75)	na	(20)
		0% (0/93)	1% (ns/93)	0% (0/93)	(22)
	Lung adenocarcinoma	ns	ns	0.005% (1/17827) FGFR4-ns	(31)
		na	0.27% (1/363)	na	(39)

Ns, not stated; Na, not analyzed.

Preclinical studies have shown that inhibition of FGF/FGFR signaling induces apoptosis in lung cancer cell lines *via* oxidative stress and impairs tumor growth in xenograft models (40, 41). The FGFR pathway may be altered by gene amplification, mRNA overexpression, and mutation and chromosomal rearrangement (gene fusion). Moreover, alternatively spliced FGFR isoforms participate in oncogenic signaling, promoting tumorigenesis.

### FGFR Gene Amplification

FGFR amplification is a main mechanism of FGFR signaling activation and is critical for cancer cell proliferation and survival. It is the most common FGFR alteration in various cancer types (reviewed in (5, 16, 22). FGFR1 amplification is frequent in Sq-NSCLC (4.6–22%, **Table 1**) and in breast cancer (5–15%), but is less common in other cancer types such as ovarian (5%) and colorectal (2%) carcinomas (4, 18, 22, 42) (**Supplemental Table S2**).

#### FGFR1 Amplification in Preclinical *In Vitro* and *In Vivo* Studies

It has been proposed to be a biomarker of FGFRi efficacy in advanced Sq-NSCLC, and preclinical *in vitro* and *in vivo* studies yielded promising results. For example, FGFRis such as PD173074 (selective FGFR1–3 inhibitor) (17, 43), AZD4547 (a potent inhibitor of FGFR1–3) (19), nintedanib (multikinase inhibitor targeting FGFR, VEGFR, and PDGFR) (20), dovitinib (multitarget inhibitor of RTKs: FLT3/c-Kit, FGFR1–3, and VEGFR1–4) (37), BGJ398 (infigratinib) (44), and ponatinib (AP24534, a multitarget pan-FGFRi) (21, 45, 46) resulted in cell growth inhibition and apoptosis of lung cancer cell lines with FGFR1 amplification (NCI-H520, LK-2, and NCI-H1703 [squamous cell carcinoma] (19–21, 40, 42–46) and NCI-H1581 [large-cell carcinoma] (17, 40, 43, 45, 46). By contrast, a squamous lung carcinoma cell line with the wild-type FGFR1 gene copy number (NCI-H2170) was insensitive to those inhibitors (17, 19, 40, 43). Preclinical *in vivo* studies based on a primary, FGFR1-amplified Sq-NSCLC xenograft revealed the therapeutic efficacy of some FGFRis. Nintedanib inhibited the growth of tumors formed by *FGFR1*-amplified NCI-H520 and LK‐2 cells (20); PD173074 shrunk xenografted FGFR1-amplified NCI-H1581 cell tumors; and AZD4547 led to significant tumor growth inhibition (TGI > 94–199%) in four of five FGFR1-amplified patient-derived squamous lung cancer models (47). However, the predictive value of FGFR amplification was not confirmed by other *in vitro* studies. Two FGFR1 non-amplified cell lines were sensitive to AZD4547 (19). An FGFR1-amplified lung cancer cell line (NCI-H520) was not sensitive to PD173074 or AZD4547 (17, 48), and FGFR1-amplified squamous lung cell lines derived from metastatic sites (HCC95 and SKMES1) were insensitive to ponatinib and AZD4547 (19, 21). Additionally, SKMES1 cells were sensitive to dovitinib (40). Nevertheless, the majority of the promising preclinical evidence resulted in FGFR1 amplification being proposed as a predictive biomarker of FGFR inhibition in advanced Sq-NSCLC.

#### FGFR1 Amplification in Clinical Trials

Consequently, it has been a key inclusion criterion in phase I/II clinical trials in solid tumors, particularly in Sq-NSCLC. However, most clinical trials in patients with Sq-NSCLC have indicated considerable weakness of FGFR amplification as a biomarker. A complete response (CR) or partial response (PR) to FGFRis has been reported for a few patients; most had stable disease (SD) or disease progression (P). In a phase Ic multicenter study of AZD4547 (NCT00979134), 1 of 24 patients with FGFR-amplified Sq-NSCLC achieved a PR (12 weeks) (49). Similarly, in a phase Ib study, 1 of 13 patients (8%) achieved PR (PFS, 5.4 months), 4 (31%) showed SD and 8 (61%) exhibited P. Changes in tumor size ranged from shrinkage by 35% to growth by 35% (23). Likewise, in a phase II lung-MAP clinical substudy (SWOG S1400D, NCT02965378), 1 of 23 patients (4.3%) with FGFR1 amplification presented a PR (2.9 months) to AZD4547 (11). More promising results were reported in a phase I clinical study of BGJ398 (NCT01004224) (10) and a phase II study of >dovitinib (NCT01861197) (9). In both studies, ~ 11% of patients with FGFR1-amplified Sq-NSCLC achieved PR (4/36 and 3/26, respectively) with a decrease in tumor size of ~ 30–40%, and ~ 40% of patients had SD. The median progression-free survival was 2.9 months and the mean duration of response was 5.2 months (9). A similar outcome was reported by basket- or umbrella-type clinical trials in FGFR1-amplified advanced cancers; Sq-NSCLC had diverse sensitivity to FGFRis; SD was the best response to LY2874455 (a pan-FGFR1–4 inhibitor that occupies the ATP-binding pocket in the kinase domain, NCT01212107) (8), Debio-1347 (CH5183284, an ATP competitive, highly selective inhibitor of FGFR1–3, NCT01948297) (50), and derazantinib (ARQ 087, an ATP-competitive pan-FGFRi with multikinase activity, NCT01752920) (51). Therefore, there is discordance between FGFR1 amplification status and the clinical response to FGFRis in Sq-NSCLC.

#### FGFR1 Focal Amplification or Arm Amplification

Failure of FGFR1 amplification as a predictive biomarker in lung cancer may be linked to expression of neighboring genes in the 8p11 amplicon. FGFR1 amplification could reflect arm-level 8p11 gain (19, 23, 40) but not focal amplification, as previously claimed (17). Initially, significant correlation between high expression of *FGFR1* and its neighboring genes [*BAG4*, *LSM1*, *NSD3* [*WHSC1L1*] (19, 40), *ASH2L*, *DDHD2*, *TACC1*, and *EIF4EBP1* (19)] and *FGFR1* amplification in Sq-NSCLC tissue was shown. However, Paik et al. (23) confirmed that expression of *FGFR1* and neighboring genes was variable and lower in tumor samples than in FGFR1-amplified cell lines. *FGFR1* expression was low in most tumor samples, but the expression patterns of other genes in the 8p11-12 amplicon were heterogeneous. In some tumors, genes in the 8p11-12 amplicon located closer to the centromere presented high expression, but in others expression levels were uniformly low, or the pattern was inconsistent. There was also no correlation between AZD4547 efficacy and 8p11 amplicon gene expression (23). This implies the importance of other genes in this locus and that FGFR1 amplification alone does not provide adequate sensitivity to reliably predict response to FGFRis.

#### FGFR1 Copy Number and mRNA Expression

Important insight into the mechanism of failure of FGFR1 amplification as a biomarker is provided by the poor correlation between *FGFR1* mRNA expression and its amplification. Only 50% of Sq-NSCLCs with an increased FGFR1 gene copy number (CGN) overexpress *FGFR1* mRNA (4). This observation was confirmed by others; 31–46% of FGFR1-amplified Sq-NSCLCs presented high *FGFR1* mRNA expression, and no *FGFR1* mRNA expression was found in 25% of FGFR1-amplified tumors (9). Similarly, Wynes et al. (21) showed that a high FGFR1 CGN overlapped with an increased *FGFR1* mRNA level in 46% of tumors (squamous/mixed NSCLC and NOS). These data imply the existence of mechanisms other than increased GCN regulating *FGFR1* transcription.

#### FGFR1 Amplification Functional Impact - Driver of Cancer or “Passenger” Event

Additionally, it remains unclear whether FGFR1 amplification represents the underlying molecular cause as a driver of cancer, or simply exists as “passenger” event within the overall mutational profile of cancer. Initially, the *FGFR1* amplification in Sq-NSCLC was hypothesized as oncogenic driver event. Functional studies revealed that silencing of FGFR1 strongly reduced the viability of the FGFR1-amplified lung cancer cells, inhibited cancer cell growth and clonogenicity, while application of the FGFR inhibitor revealed growth inhibition (p=0.0002) and induced apoptosis (p=0.008) (17, 46). FGFR1 amplification also have indicated poor prognosis, since its association with shorter overall survival and shorter disease-free survival was reported (52). Despite initial evidence, data from *in vitro*, *in vivo* and clinical studies (as discussed in dedicated sections) revealed conflicting data. Mainly, poor correlation of FGFR1 amplification with *FGFR1* gene and protein expression in Sq-NSCLC cells, and ambiguous response to FGFR1 inhibition affected either due to the *FGFR1* expression or presence of other alternations and subsequent activation of alternative signaling pathways, might indicate that FGFR1 amplification is a passenger alteration event rather than a driver alteration. Furthermore, a concurrent amplification of FGFR1 and other genes located in distant loci (on other chromosomes) has been acknowledged in Sq-NSCLC recently (27), specifically the co-amplification of FGFR1 with Defective In Cullin Neddylation 1 Domain Containing 1 gene (DCUN1D1, activation of the focal adhesion kinase and hedgehog signaling pathways) and/or BCL9 Transcription Coactivator gene (BCL9, an alternate activation of Wnt/b-catenin pathway) in the 93% FGFR1-amplified Sq-NSCLC. Still, association of this co-amplification with FGFRi treatment effect has not been examined yet.

#### *FGFR2–4* Amplification

In contrast, the reliability of *FGFR2–4* amplification (which is rare in Sq-NSCLC [0–4.7%], **Table 1**) (11, 20, 22, 36, 53) as a predictive biomarker for Sq-NSCLC has not been investigated. The only phase II clinical study (NCT02965378) of AZD4547 revealed similar inconsistencies in FGFR1 amplification. Two patients with FGFR3-amplified Sq-NSCLC have been reported: one with tumor shrinkage by 25% and the other (with concomitant FGFR1 amplification) with a 10% increase in tumor size (11).

These data imply reasons for the low predictive value of FGFR1 amplification for response to FGFRis. Unfortunately, few Sq-NSCLC patients have been treated with FGFRis in clinical trials, precluding firm conclusions. However, a number of clinical trials of FGFRi are ongoing and are actively enrolling *FGFR*-amplified patients with advanced Sq-NSCLC (**Figure 2**).

### FGFR mRNA/Protein Overexpression

*FGFRs* are overexpressed not only in lung cancer but also in other solid tumors, including cancers of the prostate, breast, brain, gastric and head and neck, and sarcoma (54, 55). Increased *FGFR1* mRNA levels are frequently observed in Sq-NSCLC. Medium and high tumor *FGFR1* mRNA expression was reported in 27–60% of patients with Sq-NSCLC (4, 19, 26), and 55 of 118 (47%) Sq-NSCLC tumors had *FGFR1–3* mRNA overexpression (55, 56).

#### FGFR mRNA Expression

As FGFR1 amplification is a poor predictive biomarker for response to FGFRis in Sq-NSCLC, *FGFR* expression levels have been evaluated in preclinical and clinical studies. *In vitro*, a high *FGFR1* mRNA level, independently of its amplification, was a significant predictor of sensitivity to AZD4547 in two FGFR1 non-amplified squamous lung cancer cell lines (H226 and LK2) compared to the H1703 and H520 lines, characterized by both high FGFR1 amplification and overexpression (19). Moreover, BGJ398 treatment of FGFR1-silenced H-520 cells showed results similar to BGJ398 or siRNA-FGFR1 treatments alone, indicating that the drug inhibitory effects actually relied on FGFR1 inhibition, while complete silencing of the FGFR1 expression in H-520 cell line lead to significant reduction of cell proliferation, increased the percentage of cells in the G_0/1_ phase of the cell cycle and resulted in down-regulation of both MAPK and AKT/mTOR pathways (44, 46). Also, the majority of Sq-NSCLC cell lines established from primary or metastatic tumors with *FGFR1* expression subsequently transfected by a dominant-negative FGFR1 (dnFGFR1) IIIc-green fluorescent protein chimera or treated with FGFR small-molecule inhibitors (SU5402 and PD166866) showed significantly reduced growth, survival, clonogenicity, and migratory potential. The FGFRi treatment had no effect on nonmalignant bronchoepithelial cell lines (BEAS-2B) expressing very low levels of *FGFR1* (57).

In addition, *FGFR3*, but not *FGFR2* or *FGFR4*, mRNA levels were weakly associated with sensitivity to ponatinib (no correlation to AZD4547) (19, 21). These promising results were confirmed *in vivo*: significant inhibition of tumor growth by rogaratinib (a potent and selective FGFR1–4 inhibitor) was observed in two patient-derived xenografts (PDX) (LU299–Sq-NSCLC and LXFL1121-NSCLC) overexpressing *FGFR1* mRNA but not harboring high *FGFR* amplification (58). Also, two differentially expressed gene profiles of Sq-NSCL tumors (PDX), sensitive or resistant to dovitinib were observed (40). Notably, *FGFR3*, *FGF3* and *FGF19* were the most diversely expressed genes, and significant differences in *GRB2*, *KLB*, *MAPK1*, *NRAS*, and *SOS1* expression were reported. The different expression patterns encompassed genes related to the FGFR pathway, including FGFR1–4 receptors but also other oncogenic genes, which might be linked to tumor sensitivity to FGFRis. The results of phase II clinical studies are inconsistent. Promising results were obtained in 40 advanced Sq-NSCLC patients with *FGFR1-3* overexpression (NCT03762122) treated with rogaratinib; two patients (5.6%) achieved PR (in one, for > 16 months) and SD was achieved in 64% (51, 59). However, only 1 of 10 patients (1%) had 6 months PFS in continuation of that study (60). Also, in three Sq-NSCLC patients with enhanced *FGFR1* mRNA levels, two responded to ponatinib with SD and one showed P (61). In contrary, in one of three dovitinib responders in the NCT01861197 trial, low *FGFR1–3* mRNA levels and a high FGFR1 amplification level in the tumor were reported (9).

#### FGFRs Protein Expression

FGFR1-3 protein levels are frequently high in NSCLC. Notably, FGFRs expression level differs between histological subtypes. A high FGFR1 protein level was reported in: Sq-NSCLCs - 16 of 171 (9%) (24), 13 of 212 (6%) (26), and in 22 of 267 (8.2%) (36); adenocarcinoma histological subtype- 40 of 114 (35%) (24), 13 of 383 (3.5%) (26), and 40 of 309 (12.9%) (36). High FGFR2 and FGFR3 protein levels in were also reported in both NSCLC histotypes: Sq-NSCLC: 9 (3.4%) and 18 (6.6%), respectively; -adenocarcinoma: 66 (21.8) and 2 (0.6%), respectively (36). Nonetheless, there was no significant correlation between FGFR1 protein expression and gene amplification assessed by FISH or NGS (23, 24), although there was a weak correlation between FGFR1 mRNA and protein levels in Sq-NSCLC (19, 26). The FGFR protein levels have also been investigated as potential biomarkers for response to FGFRi treatment. Some preclinical studies have indicated that FGFRi response associates closely with high FGFR1 protein expression. For example, Wynes et al. (21) showed that FGFR1 mRNA and protein expression, but not GCN, predict FGFR TKI sensitivity in lung cancer cell lines. Similarly, use of rogaratinib against cancer cell lines with high FGFR expression but no FGFR amplification revealed the selective antiproliferative activity of FGFRis (58). On the other hand, two *FGFR1*-amplified NSCLC PDX (from large cell and adenocarcinoma subtypes) with co-overexpression of FGFR1 protein responded to M6123 (selective FGFR1 antagonist) (26). To our knowledge, besides one Sq-NSCLC patient with FGFR1 amplified tumor, who achieved PR in NCT00979134 study and had no FGFR1 protein overexpression (23) there is luck of clear results for clinical significance of FGFR1 protein expression. Because of the dearth of clinical data, the relationship between FGFR mRNA/protein levels and response to FGFRis remains unclear. On-going trials of FGFRis are enrolling *FGFR* mRNA- and protein- overexpressing patients with advanced Sq-NSCLC (**Figure 2**).

### FGFR Mutations and Fusions

#### *FGFRs* Mutations

*FGFR* mutations are frequent in human cancers (16, 22), with the highest prevalence in NSCLC (*FGFR1* range 0–18%) (17, 20, 23, 62), endometrial carcinoma (*FGFR2*, range 0–9%) (35, 63), bladder carcinoma (*FGFR3*, range 8.5–26%) (53, 64), and rhabdomyosarcoma (*FGFR4* 7.5%) (65) (**Table 1** and **Supplemental Table S2**). Mutations are found in the regions of the gene encoding the extracellular, trans-membrane, and kinase domains of the FGFR receptor (**Figure 1** and **Tables 2** and **3**) but are most frequent outside the kinase domain in exons corresponding to the extracellular domains (Ig-domains) and alternative exons.

**Table 2:** *FGFR1-2* variants detected in squamous non-small cell lung cancer (Sq-NSCLC). Variant localisation is shown in **Figure 2**.

Gene name	Localization		Variants		Variant origin/Functional effect of alteration	Clinical significanceClinVar/VarSome Clinical**	Source
**FGFR1**	extracellular domain	alternative exon	c.80C>T*	T27I*	somatic/unknown	Not reported/Uncertain Significance	(20)
		Ig I	c.208G>A	G70R	somatic/unknown	Likely pathogenic​/Pathogenic	(20)
		alternative exon	c.277G>T*	D93Y*	somatic/unknown	Not reported/Uncertain Significance	(23)
			c.391G>A	D131N	somatic/unknown	Uncertain Significance/Uncertain Significance	(23)
			c.410A>G (c.509A>G)*	E137G	somatic/unknown	Not reported/Likely Pathogenic	(62)
			c.749G>C (c.842G>C)*	R250P	somatic/unknown	Likely pathogenic​/Likely Pathogenic	(62)
			1107G>A (c.1200G>T)*	M369I	germline/unknown	Not reported/Uncertain Significance	(62)
	cytoplasmic domain		c.1366A>G (c.1459A>G)*	M456V	somatic/activating	Not reported/Likely Pathogenic	(62)
		kinase domain	c.1600A>G (c.1693A>G)*	M534V	germline/unknown	Not reported/Benign	(62)
			c.2383G>A (c.2476G>A)*	V795I	germline/unknown	Not reported/Uncertain Significance	(62)
**FGFR2**	extracellular domain		c.41C>T	T14I	Somatic /unknown	Not reported/Uncertain Significance	(20)
		Ig II	c.632A>T	N211I	Somatic/not stated	Not reported/Likely Pathogenic	(35)
			c.755C>G	S252W	somatic/activating	Pathogenic​/Pathogenic	(22, 34),
			c.758C>G	P253R	somatic/activating	Pathogenic​/Pathogenic	(22)
		Ig III	duplication of 21 bp	A266_S267ins STVVGGD	somatic/oncogenic	Not reported/Not Reported	(66)
		Ig III	c.870G>T c.870G>C	W290C	somatic/activating	Pathogenic​/ Pathogenic	(34, 35, 62)
		Ig III alternative exon	c.959C>G	S320C	Not stated/activating	Likely pathogenic​/Likely Pathogenic	(34)
	cytoplasmic domain		c.1199G>A	R400Q	Not stated/unknown	Not reported/Likely Pathogenic	(23)
			c.1216A>G	K405E	germline/unknown	Conflicting interpretations of pathogenicity/Uncertain Significance ​	(62)
		kinase domain	c.1744C>T	P582S	somatic/unknown	Not reported/Likely Pathogenic	(20)
		kinase domain	c.1978A>G	K660E	not stated/activating	Likely pathogenic/Likely Pathogenic​	(34, 67)
		kinase domain	c.1980G>C	K660N	not stated/activating	Pathogenic​/Likely Pathogenic	(34, 67)
		kinase domain	c.1885G>A (c.1534G>A)*	A629T (A511T)*	germline/unknown	Pathogenic/Likely Pathogenic​	(62)
			c.2390C>A	S797Y	somatic/unknown	Not reported/Uncertain Significance	(62)

Reference transcripts: FGFR1: NM_023110.3, NP_075598.2 (*NM_001174067.1, NP_001167538.1); FGFR2: NM_022970.3, NP_075259.4, *(NM_001144917.2, NP_001138389.1); FGFR3: NM_000142.4, NP_000133.1,*(NM_001163213.1, NP_075254); FGFR4: NM_213647.3, NP_075252, *(NM_022963.3).

**Clinical significance based on ClinVar and VarSome databases.

**Table 3:** *FGFR3-4* variants detected in squamous non-small cell lung cancer (Sq-NSCLC). Variant localisation is shown in **Figure 2**.

Gene name	Localization		Variants		Variant origin/Functional effect of alteration	Clinical significanceClinVar/VarSome Clinical**	Source
**FGFR3**	extracellular domain	Ig I	c.87G>C	Q29H	germline/unknown	Not reported/Uncertain Significance	(62)
			c.403_405delGAA	E135del	germline/unknown	Not reported/not reported	(62)
		Ig II	c.490C>G	L164V	germline/unknown	Likely benign​ /Likely benign	(62)
			c.742C>T	R248C	somatic/activating	Pathogenic​/Pathogenic ​	(22, 34, 67)
			c.746C>G	S249C	somatic/activating and transforming	Pathogenic/Likely Pathogenic ​	(10, 18, 22, 23, 34, 39, 62, 67)
			c.1108G>T	G370C	somatic/activating	Pathogenic/Pathogenic	(22)
			c.1118A>G	Y373C	germline/activating	Pathogenic/Pathogenic	(67)
	transmembrane domain		c.1138G>A	G380R	not stated/unknown	Pathogenic/Not reported	(67)
	cytoplasmic domain	kinase domain	c.1498G>A (c.1504G>A)*	A500T	somatic/unknown	Not reported/Uncertain Significance	(62)
		kinase domain	c.1587G>T (c.1593G>A)*	M529I	germline/unknown	Not reported/Likely Pathogenic	(62)
		kinase domain	c.1748A>T (c.1754A>T)*	K583M	germline/unknown	Not reported/Likely Pathogenic	(62)
		kinase domain	c.1948A>G	K650E	somatic/activating	Pathogenic​/Pathogenic	(22)
		kinase domain	c.2144A>T (c.2150A>T)*	K717M	Not stated/Not activating	Not reported/Likely Pathogenic	(34)
			c.2380C>G (c.2386C>G)*	L794V	germline/unknown	Not reported/Uncertain Significance	(62)
**FGFR4**	extracellular domain	Ig I	c.146T>A	L49H	germline/unknown	Not reported/Likely Benign	(62)
		Ig II	c.686C>T	A229V	germline/unknown	Not reported/Likely Benign	(62)
	transmembrane domain	alternative exons	c.1141G>A*	E381K	somatic/ unknown	Not reported/Uncertain Significance	(20)
		alternative exons	c.1145C>T*	S382L	somatic/ unknown	Not reported/Uncertain Significance	(20)
	cytoplasmic domain	alternative exons	c.1145C>T*	G408S	somatic/ unknown	Not reported/Uncertain Significance	(20)
		kinase domain	c.1727G>A	G576D	germline/unknown	Not reported/Likely Benign	(62)
			c.2266G>T	D756Y	somatic/unknown	Not reported/Uncertain Significance	(62)

Reference transcripts: FGFR1: NM_023110.3, NP_075598.2 (*NM_001174067.1, NP_001167538.1); FGFR2: NM_022970.3, NP_075259.4, *(NM_001144917.2, NP_001138389.1); FGFR3: NM_000142.4, NP_000133.1,*(NM_001163213.1, NP_075254); FGFR4: NM_213647.3, NP_075252, *(NM_022963.3).

**Clinical significance based on ClinVar and VarSome databases.

This is important because point mutations in extracellular domains lead to receptor stimulation in a ligand-independent manner by obligate receptor dimerization (68). For example, S249C mutation in the extracellular domain (IgIII) of *FGFR3* induces constitutive dimerization and receptor activation *via* modest dimer stabilization in the absence of ligand (69). Similarly, rare *FGFR* mutations in the transmembrane domain may reduce dimerization efficiency and overall stability, leading to a constitutively active protein (70). Kinase-domain point mutations are typically localized in regulatory elements such as the molecular brake, the A-loop, the kinase hinge, and the DFG-latch. This results in receptor autophosphorylation and constitutive receptor activation in a ligand-independent manner, as exemplified by oncogenic-like *FGFR2* mutations (A266_S267ins, 290_291WI>C) (66) and point mutations in *FGFR3* (N540, K650) (68).

#### FGFRs Fusions

*FGFR* gene fusions originating from chromosomal rearrangement of two genes (through translocation, insertion, inversion or deletion) are found in all common tumor types. The Cancer Genome Atlas study reported *FGFR* fusions in 8 of 20 tumor types (29). Chromosomal rearrangements involving *FGFR* genes often lead to protein fusion with the FGFR kinase domain and its subsequent activation (71). *FGFR2* gene fusions are most frequent in cholangiocarcinoma, and are also detected, albeit less commonly, in colorectal cancer, hepatocarcinoma and NSCLC (22). *FGFR3* fusions are more common in glioblastoma, urothelial carcinoma, and lung cancer, with several dozen fusion partners. *FGFR1* and *FGFR4* fusions are rare in solid tumors. *FGFR1* fusions are typically found in gastrointestinal stromal tumor (GIST), breast cancer, and bladder urothelial carcinoma. Lung cancer develops *via* a multistep process of tumor biogenesis involving accumulation of inherited or acquired genetic abnormalities. Analysis of the *FGFR* alterations in Sq-NSCLC showed that 20% of identified variants were in *FGFR1-4* (20, 34, 62) (**Table 2** and **3**); FGFR1 ~ 18% (17, 20, 23, 62), *FGFR2* 2.5–4.7% (20, 34, 35), *FGFR3* 0–9% (20, 23, 34), and *FGFR4* 5.3% (20). In addition, 0.6–3.5% of FGFR aberrations are chromosomal translocations such as FGFR3-TACC3 (3.5%), BAG4-FGFR1 (< 1%), and FGFR2-KIAA1967 (0.3%) (31, 72). Fusions of all four *FGFR* genes have been found in Sq-NSCLC (30, 31, 67, 71, 73).

#### Importance of FGFRs Mutations and Fusions as FGFRi Predictive Biomarkers

FGFR mutations and fusions are candidate predictive biomarkers for FGFRis. For instance, it was recently shown that estimated drug sensitivity of *FGFR3* S249C was intermediate for all FGFR inhibitors examined in mouse fibroblast cell line (3T3 cells), while different variants at *FGFR2* N549 showed different drug sensitivity but similar oncogenicity (74). However, there is little evidence for the predictive power of FGFR variants in Sq-NSCLC. Contradictory results from AZD4547 trials in patients with Sq-NSCLC with the *FGFR3* S249C pathogenic variant have been reported. The phase II lung-MAP substudy (SWOG S1400D, NCT02965378) and a phase Ib clinical study (NCT00979134) with AZD4547 FGFRi revealed single Sq-NSCLC patients with *FGFR3* S249C who achieved PR (for 1.5 months with ~ 32% tumor shrinkage) (11) *or SD* (2.6-month progression-free survival [PFS] and 12% tumor shrinkage) (23). No significant benefit of AZD4547 (11) or BGJ398 (infigratinib, NCT01004224) (10) was shown in two other patients carrying the *FGFR3* S249C variant (*2–4%* tumor size decrease). Diverse treatment outcomes were observed in Sq-NSCLCs with other *FGFR* variants: SD (4.1 months PFS and 20% tumor *shrinkage*) was reported in one patient (6.6%) with simultaneous *FGFR1* missense variants: D93Y and H841Y (23), whereas no response was reported in a patient (1/27, 1.4%) with *FGFR3* fusion (11). Additionally, the objective response rate was observed in 1 (5%) patient carrying the *FGFR* mutation or fusion among 24 patients with NSCLC (13%) treated with erdafitinib (NCT01703481) (75). Molecular profiling revealed 10 (42%) *FGFR* mutations, 8 (33%) fusions, and 5 (21%) amplifications in that group. FGFR variants and fusions may have predictive utility in other tumor types such as bladder/urothelial cancer, cholangiocarcinoma, and adrenocortical carcinoma (NCT01004224, NCT01752920, NCT02465060) (10, 47, 76, 77). Abnormal protein conformation resulting from FGFR aberrations, and consequently ineffective FGFRi binding, may be responsible for the weak predictive power of FGFR variants and fusions for response to FGFRis (78). Functional analysis can identify potential associations of rare and newly identified variants with FGFR activation, with later confirmation by transforming activity *in vitro*. Additionally, other factors such as pre-existing mutations of neighboring genes might also influence FGFRi binding. The scarcity of available data, mostly from individual Sq-NSCLC cases as well as a few clinical studies, indicates the need for further research. A number of ongoing clinical trials involve patients with advanced Sq-NSCLC and FGFR mutations and/or translocations (**Figure 2**).

## Potential Markers of FGFRi Resistance

Anti-FGFR therapeutic strategies based on small-molecule chemical tyrosine kinase inhibitors (TKIs) are by far the most extensively explored. However, in clinical and preclinical trials, cancer cells developed diverse resistance mechanisms to FGFRis (5).

### *FGFRs* Mutations

*FGFR* secondary mutations at the gatekeeper residue and gene fusions are the main mechanisms of resistance to FGF/FGFRis. For instance, the V561M *FGFR1* gatekeeper mutation caused resistance to AZD4547 *via* STAT3 activation and the epithelial-mesenchymal transition in H1581 cells (79) and prolonged exposure of KMS-11 multiple myeloma cells (*FGFR3* Y373C) to AZD4547 resulted in a second *FGFR3* kinase domain point mutation (V555M), triggering a conformational change and preventing adequate drug binding (80). Additionally, the3T3 cells with *FGFR1* N546K and *FGFR2* N549D/K were resistant to AZD4547, BGJ398 (infigratinib), erdafitinib, and pemigatinib, and simultaneously they were relatively sensitive to E7090 (a potent selective FGFR1‐3 inhibitor) and futibatinib (selective, irreversible FGFR1–4 inhibitor) (74). The authors also showed that the3T3 cells with concurrent mutations in *FGFR3* (S249C and K650M) were greatly resistant to E7090 in comparison to cells with individual mutations. However, such a strong association was not demonstrated for Erdafinib. The *FGFR2* mutation V564F in the kinase domain conferred BGJ398 (infigratinib) resistance in cholangiocarcinoma *FGFR2* fusion-positive patients (81) and the *FGFR2-ASCL5* fusion (Ig2, I-set, a tyrosine kinase domain from FGFR2, and a truncated AMP-binding domain from ACSL5) was reported in post-progression biopsy of a patient with FGFR2-amplified gastric cancer with prior response to the FGFRi LY2874455 (82). FGFR structure analysis has shown that some FGFR gatekeeper mutations lead to steric clashes with FGFRis (78). Consequently, shifting the drug-binding site from the mutation site can overcome resistance to LY2874455, despite FGFR gatekeeper mutations (*FGFR1* (V561M), *FGFR2* (V564F), *FGFR3* (V555M), and *FGFR4* (V550M and V550L) (78).

### Activation of Multiples Signaling Pathways

PI3K–AKT signaling mediates resistance to FGFRis by directly affecting cell proliferation or activating the mTOR pathway, altering cell metabolism and anti-apoptotic signaling (83). Recently, the dysregulation of RAS/RAF/MAPK pathway through *MAPK14* gene (Mitogen-Activated Protein Kinase 14, p38 MAPK) was shown as a driver of resistance to a novel FGFR inhibitor CPL304110 (NCT04149691) (84). Authors revealed that activation of p38 kinase resulted in resistance to CPL304110 in parental cells, while inhibition of p38 MAPK resensitised resistant cells to that FGFRi treatment. Other receptor tyrosine kinases such as hepatocyte growth factor receptor (MET/HGFR) may be upregulated in response to FGFRis, thereby serving as a bypass mechanism for activation of signaling (85, 86). Data from preclinical studies, including cellular models of lung, colorectal, and gastric cancers, indicate that the MET pathway is vital for the growth, survival, and invasive potential of cancers (87). However, little is known of primary resistance to FGFRis. Primary resistance mechanisms may be related to alterations unique to *FGFR*s and *MET*s. Hepatocyte growth factor (HGF) binding to MET induces activation of various downstream signaling pathways, including the RAS/MAPK and PI3K/AKT pathways, leading to cell proliferation, survival, and migration (87). *MET* amplification is an important mechanism by which cancers develop resistance to epidermal growth factor receptor (EGFR) inhibitors and FGFRis, and a high *MET* GCN has been detected in 1–11% of NSCLCs. *MET* amplification may lead to secondary resistance to EGFR-TKIs in patients with *EGFR*-mutated lung adenocarcinoma (88). However, MET activation is not observed solely in the presence of amplification, implying other mechanisms of signaling activation (89). These may depend on the molecular aberrations of the *MET* gene altering *MET* transcription, MET translation or degradation, or may directly transactivate MET receptor. In 3–4% of NSCLCs, *MET* splice mutations explained exon 14 skipping, leading to impaired MET degradation (82). Additionally, *in vitro* models of FGFRi-resistance confirmed a role for AKT and MET activation and clonal expansion of the S266L *AKT1* mutation (86). An improved understanding of the complex mechanisms of FGFRi resistance in lung cancer is needed.

## Perspectives

Most *in vitro* studies have demonstrated the potential of *FGFR* aberrations to predict sensitivity to FGFRis. Unfortunately, clinical data are scarce and discordant.

Important obstacle to patient selection is the availability of archival tumor samples for molecular analysis. Liquid biopsy has emerged as a potential diagnostic approach. Cell-free DNA (cfDNA) analysis is a non-invasive method routinely used in the clinical setting to evaluate secondary resistance mutations to TKIs [reviewed in (90)]. The *FGFR* alterations detected in cfDNA however, show discordance with tumor molecular profiles. A retrospective analysis of 17 cholangiocarcinoma cases revealed *FGFR2-3* alterations in 82.3% of archival tumour samples and 50% of DNA samples (4/5 SNVs, 1/2 amplifications, and 5/13 fusions) (91). Likewise, *FGFR2* fusions were detected in the primary tumor and cfDNA in 8 of 12 (67%) patients prior to BGJ398 treatment (NCT02150967) (92). cfDNA has potential for analysis of FGFR2 amplification in gastric and breast cancers. In Sq-NSCLC, cfDNA enabled monitoring of copy number variation and identified a 71% overlap of *PIK3CA* gain in cfDNA and tumour tissue (phase II clinical trial, GO27912, NCT01493843 (93). cfDNA also has potential for continuous monitoring of the molecular response and resistance mechanisms, as for EGFR TKIs (94). Accordingly, in three patients with cholangiocarcinoma treated with BGJ398 (phase II study, NCT02160041) the FGFR2-BICC1 fusion decreased in cfDNA upon initiation of treatment and increased at the time of radiological progression (81). *FGFR2* mutations that potentially confer resistance (V564L, V564F) and activation (N549K) together with alterations in other candidate resistance genes (*PTEN* and *MAP2K1*) and a low variant allele frequency of *NRAS* G12D and *BRAF* A694T were detected in cfDNA samples from patients on FGFRis who experienced disease progression (91). Notably, because high *FGFR* mRNA levels were predictive of responsiveness to FGFRis, cell-free RNA (cfRNA) may be an alternative to tissue-based gene-expression analysis. This approach is, however, technically challenging because of the potential for sample contamination with white-blood-cell mRNA. Also, stable normalization transcripts in plasma are unknown, although some results are promising (95).

## Discussion

FGFR genomic aberrations facilitate a response to FGFRis, prompting a search for reliable predictive biomarkers. Unfortunately, despite the promising data from preclinical and clinical studies of FGFRis, we are far from identifying specific aberration(s) in FGFRs. *FGFR* mRNA overexpression, mutations, and fusions show promise, but their discriminatory power is insufficient. Despite the FGFR aberrations in Sq-NSCLC tumors (alterations: mutations and fusions; amplification; mRNA/protein overexpression), the association between the molecular profile and the response to FGFRi treatment is unclear. Small clinical trials of FGFRis in Sq-NSCLC have yielded little data. Moreover, most clinical trials have been of the basket-type design and enrolled small numbers of patients with Sq-NSCLC. Additionally, genomic aberrations other than FGFR could affect the response to FGFRis.

In conclusion, identification of clinically relevant predictive biomarkers of the response and/or secondary resistance to FGFRis in Sq-NSCLC patients is challenging. There is an urgent need for basic and clinical research based on squamous lung cancer histology and deep DNA/RNA profiling to provide insight into cancer biology and to promote discovery of candidate biomarker(s).

## Author Contributions

All authors were involved in the concept and design. JM-S wrote the manuscript. JC-W critically reviewed the manuscript. All authors read and approved the final manuscript.

## Funding

This work was supported by STRATEGMED2 grant (STRATEGMED2/266776/17/NCBR/2015, project “CELONKO”).

## Conflict of Interest

JC-W disclose grants, personal fees or non-financial support outside the submitted work from: Grifols, AstraZeneca, MSD, BMS, GSK, Novartis, Chiesi, Roche, Boehringer, CSL Behring, CelonPharma, Amgen, Lekam, Mereo, Takeda.

The remaining author declares that the research was conducted in the absence of any commercial or financial relationships that could be construed as a potential conflict of interest.

## Publisher’s Note

All claims expressed in this article are solely those of the authors and do not necessarily represent those of their affiliated organizations, or those of the publisher, the editors and the reviewers. Any product that may be evaluated in this article, or claim that may be made by its manufacturer, is not guaranteed or endorsed by the publisher.
