# Supplementary material for: Fibroblast Growth Factor Receptor 1-4 Genetic Aberrations as Clinically Relevant Biomarkers in Squamous Cell Lung Cancer
Source: Front Oncol. 2022 Mar 25;12:780650. doi: 10.3389/fonc.2022.780650 (PMC8991910; doi:10.3389/fonc.2022.780650)
Supplement: Supplementary file 1 [file DataSheet_1.pdf]

Supplementary Material

1. Supplementary Figures

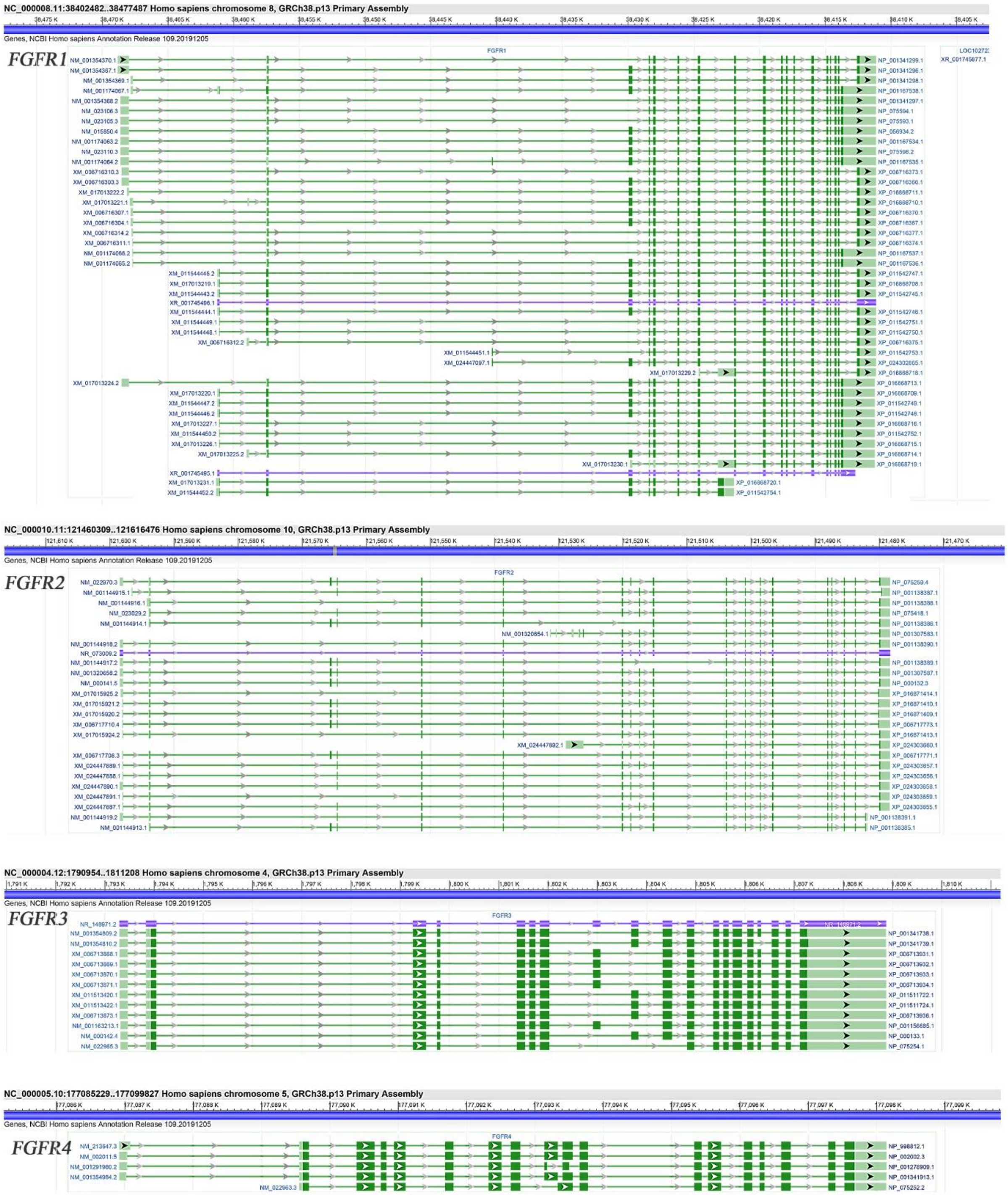

Supplementary Figure S1. Schematic overview of *FGFR1-4* gene differential splicing isoforms (NCBI)

Moes-Sosnowska J and Chorostowska-Wynimko J (2022) “Fibroblast Growth Factor Receptor 1-4 Genetic Aberrations as Clinically Relevant Biomarkers in Squamous Cell Lung Cancer”. Front. Oncol. 12:780650. doi: 10.3389/fonc.2022.780650

2. Supplementary Tables

Supplementary Table S1. *FGFR1-4* gene characteristic

Gene	Chromosome location	Gene size (bases)	Isoform name	Isoform tissue specific[1]	FGFs activation[2]	High levels of FGFR proteins in normal human adult tissues[3]	Presence of FGFR protein in lung tissue[3]
FGFR1	8 (8p12)	57,697	FGFR1b	epithelial tissue	FGF-1, 2, 3, 10	skin, cornea, lung, heart, placenta, kidney, and ureter.	+
			FGFR1c	mesenchymal tissue	FGF-1, 2, 4, 5, 6		
FGFR2	10 (10q26.13)	120,327	FGFR2b	epithelial tissue	FGF-1, 3, 7, 10	prostate and stomach	+
			FGFR2c	mesenchymal tissue	FGF-1, 2, 4, 6, 9		
FGFR3	4 (4p16.3)	15,574	FGFR3b	epithelial tissue	FGF-1,9	appendix, colon, liver, sublingual gland, placenta, and cervix	+
			FGFR3c	mesenchymal tissue	FGF-1, 2, 4, 8, 9		
FGFR4	5 (5q35.2)	11,273	-		FGF-1, 2, 4, 6, 8, 9	liver, sublingual gland ducts, kidney, and ureter, medium of some (but not all) arterioles and veins in most tissues.	

Table S1 references:

1. Beenken A, Mohammadi M. The FGF family: biology, pathophysiology and therapy. Nat Rev Drug Discov 2009; 8:235–53.

2. Ornitz DM, Itoh N. The Fibroblast Growth Factor signaling pathway. Wiley Interdiscip Rev Dev Biol 2015; 4:215–66.

3. Hughes SE. Differential expression of the fibroblast growth factor receptor (FGFR) multigene family in normal human adult tissues. J Histochem Cytochem 1997; 45:1005–19.

Supplementary Table S2. Frequency of *FGFR1-4* genes amplification and mutations among different cancers except lung cancer (showed in main manuscript)

Gene name	Type of cancer	Amplification frequency (%)	Mutations frequency (%)	Source
FGFR1	Breast cancer HER2-negative	18%		(1)
	Breast cancer	14%		(2)
	Breast cancer	10%		(3)
	Breast cancer hormone receptor-positive	15%		(4)
	Breast cancer triple-negative	5%		(4)
	Urothelial carcinoma	7%		(2)
	Small-cell lung carcinoma	8%		(5)
	Ovarian cancer	5%		(2)
	Head and neck squamous cell carcinoma HPV-negative	3%		(6)
	Rhabdomyosarcoma	3%		(4)
	Pancreatic ductal adenocarcinoma	2.6%		(7)
	Gastric/gastroesophageal junction carcinoma	2%		(2)
	Colorectal carcinoma	2%		(2)
	Carcinoma of unknown primary	2%		(2)
	Squamous non-lung tumors	2%		(2)
	Squamous differentiated bladder cancer	0%		(8)
	Glioblastoma		10.5% (2/19)	(9)
FGFR2	Gastric cancer	5%		(1)
	Gastric cancer	4.1%		(10)
	Breast cancer Triple negative	4%		(4)
	Bladder cancer squamous differentiated	0%		(8)
	Endometrial cancer		16% (18/115)	(11)
	Endometrial cancer		12% (15/122)	(12)
	Uterine tumors		10% (19/187)	(11)
	Cervical Carcinoma		4.3% (2/42)	(12)
FGFR3	Bladder cancer squamous differentiated	0%	8.5% (6/71)	(8)
	Urothelial carcinoma of the bladder		26% (44/170)	(13)
	Urothelial carcinoma		5.7% (9/159)	(14)
	Urothelial carcinoma	3%	15% (/126)	(2)
FGFR4	Rhabdomyosarcoma		7.5% (7/94)	(15)

Table S2 references:

1. Pearson A, Smyth E, Babina IS, Herrera-Abreu MT, Tarazona N, Peckitt C, et al. High-Level clonal FGFR amplification and response to FGFR inhibition in a translational clinical trial. Cancer Discov 2016; 6:838–51. https://doi.org/10.1158/2159-8290.cd-15-1246

2. Helsten T, Elkin S, Arthur E, Tomson BN, Carter J, Kurzrock R, et al.. The FGFR landscape in cancer: Analysis of 4,853 tumors by next-generation sequencing. Clin Cancer Res 2016; 22:259–67. https://doi.org/10.1158/1078-0432.CCR-14-3212

3. Turner N, Pearson A, Sharpe R, Lambros M, Geyer F, Lopez-Garcia MA, et al. FGFR1 amplification drives endocrine therapy resistance and is a therapeutic target in breast cancer. Cancer Res 2010; 70:2085–94. https://doi.org/10.1158/0008-5472.CAN-09-3746

4. Turner N, Grose R.. Fibroblast growth factor signalling: from development to cancer. Nat Rev Cancer. 2010;10(2):116–29. https://doi.org/10.1038/nrc2780

5. Elakad O, Lois A-M, Schmitz K, Yao S, Hugo S, Lukat L, et al. Fibroblast growth factor receptor 1 gene amplification and protein expression in human lung cancer. Cancer Med 2020; 9:3574–83. https://doi.org/10.1002/cam4.2994

6. Koole K, Brunen D, van Kempen PMW, Noorlag R, de Bree R, Lieftink C, et a. FGFR1 is a potential prognostic biomarker and therapeutic target in head and neck squamous cell carcinoma. Clin Cancer Res 2016; 22:3884–93. https://doi.org/10.1158/1078-0432.ccr-15-1874

7. Lehnen NC, von Mässenhausen A, Kalthoff H, Zhou H, Glowka T, Schütte U, et al. Fibroblast growth factor receptor 1 gene amplification in pancreatic ductal adenocarcinoma. Histopathology 2013; 63:157–66. https://doi.org/10.1111/his.12115

8. Baldia PH, Maurer A, Heide T, Rose M, Stoehr R, Hartmann A, et al. Fibroblast growth factor receptor (FGFR) alterations in squamous differentiated bladder cancer: a putative therapeutic target for a small subgroup. Oncotarget 2016; 7:71429–39. https://doi.org/10.18632/oncotarget.12198

9. Rand V, Huang J, Stockwell T, Ferriera S, Buzko O, Levy S, et al. Sequence survey of receptor tyrosine kinases reveals mutations in glioblastomas. Proc Natl Acad Sci U S A 2005; 102:14344–9. https://doi.org/10.1073/pnas.0507200102

10. Matsumoto K, Arao T, Hamaguchi T, Shimada Y, Kato K, Oda I, et al. FGFR2 gene amplification and clinicopathological features in gastric cancer. Br J Cancer 2012; 106:727–32. https://doi.org/10.1038/bjc.2011.603

11. Pollock PM, Cancer Genome Project, Gartside MG, Dejeza LC, Powell MA, Mallon MA, et al. Frequent activating FGFR2 mutations in endometrial carcinomas parallel germline mutations associated with craniosynostosis and skeletal dysplasia syndromes. Oncogene 2007; 26:7158–62. https://doi.org/10.1038/sj.onc.1210529

12. Dutt A, Salvesen HB, Chen T-H, Ramos AH, Onofrio RC, Hatton C, et al. Drug-sensitive FGFR2 mutations in endometrial carcinoma. Proc Natl Acad Sci U S A 2008; 105:8713–7. https://doi.org/10.1073/pnas.0803379105

13. Gust KM, McConkey DJ, Awrey S, Hegarty PK, Qing J, Bondaruk J, et al. Fibroblast growth factor receptor 3 is a rational therapeutic target in bladder cancer. Mol Cancer Ther 2013; 12:1245–54. https://doi.org/10.1158/1535-7163.mct-12-1150

14. Tomlinson DC, Baldo O, Harnden P, Knowles MA. FGFR3 protein expression and its relationship to mutation status and prognostic variables in bladder cancer. J Pathol 2007; 213:91–8. https://doi.org/10.1002/path.2207

15. Taylor JG 6th, Cheuk AT, Tsang PS, Chung J-Y, Song YK, Desai K, et al. Identification of FGFR4-activating mutations in human rhabdomyosarcomas that promote metastasis in xenotransplanted models. J Clin Invest 2009; 119:3395–407. https://doi.org/10.1172/JCI39703
